# The synergistic effects of humic acid, chitosan and *Bacillus subtilis* on tomato growth and against plant diseases

**DOI:** 10.3389/fmicb.2025.1574765

**Published:** 2025-04-17

**Authors:** Cunpu Qiu, Ying Bao, Dingding Lü, Mengyuan Yan, Guilong Li, Kai Liu, Shiping Wei, Meng Wu, Zhongpei Li

**Affiliations:** ^1^Zhenjiang College, Zhenjiang, China; ^2^State Key Laboratory of Soil and Sustainable Agriculture, Institute of Soil Science, Chinese Academy of Sciences, Nanjing, China; ^3^University of Chinese Academy of Sciences, Beijing, China; ^4^Xuzhou Vacational College of Bioengineering, Xuzhou, China; ^5^Jiangsu Vocational College of Agriculture and Forestry, Zhenjiang, China

**Keywords:** humic acid, chitosan, *Bacillus subtilis*, tomato growth, plant diseases

## Abstract

Understanding the effects of bio-antimicrobial materials on plant growth and against diseases and the relevant mechanisms are highly important for sustainable soil use and plant safety production. This study explored the impacts and corresponding mechanisms of the combined utilization of humic acid, chitosan, and *Bacillus subtilis* (*B. subtilis*) on tomato growth and diseases occurrence through a greenhouse pot experiment. The plant height, fresh weight, disease index, rhizosphere microbial community, and root exudates composition of tomatoes were determined. With the combined application of humic acid, chitosan and *B. subtilis* (HBC), the height and fresh weight of tomato plants were significantly increased (*p* < 0.05), and the incidence of plant diseases was decreased by 45.1%. In HBC treatment, the diversity of fungal and bacterial communities was notably enhanced. The relative abundances of *Bacillus, Gemmatimonas, Neobacillus, Acinetobacter, Humicola* increased, while the relative abundances of *Sphingomonas,* especially soil-borne plant pathogen *Fusarium* and *Ralstonia,* significantly decreased (*p* < 0.05). Besides the increased diversity of root exudates, the content of phenolic acids, which are allelochemicals related to continuous cropping disorder, decreased. The results of cooccurrence network analysis indicated that the abundances of Eicosanoids, Fatty acids and conjugates, and Flavonoid lycosides compounds in root exudates, which are positively correlated with pathogenic bacteria, decreased in HBC treatment. Results indicated HBC’s synergistic effect on tomato growth and disease resistance is related to its regulation of microbial community and root exudates. The study results promote the development of biological control technology and highlight its promising application in plant safety production.

## 1 Introduction

Sustainable management of plant diseases plays a crucial role in ensuring food security. The facility cultivation of tomato (*Solanum lycopersicon* L.), which is marked by intensity and efficiency, constitutes an important means to guarantee the supply of fresh products and achieve efficient production of tomatoes (Jin et al., 2024). However, due to the particular ecological environment of facility vegetable plots and the substantial input of fertilizers and pesticides, the ecological balance of the soil in facility vegetable plots has been severely disrupted, leading to disturbances in the soil microflora, the accumulation of pathogenic microorganisms, and the frequent occurrence of soil-borne diseases such as wilt (Meshram and Adhikari, 2024; Yan et al., 2025). This seriously affected the sustainable development of the facility tomato industry. Historically, several management strategies have been implemented to manage tomato soil-borne diseases, including the utilization of resistant cultivars, chemicals (e.g., fungicides and soil fumigants), physical methods such as soil solarisation and soil heating, cultural methods (e.g., crop rotation field sanitation), and biological control (Spadaro and Gullino, 2005; Meshram and Adhikari, 2024; Kashyap et al., 2023). Among these management strategies, chemical control remains the main measure. However, improper use of chemical pesticides not only reduces the efficacy, but also increases the tolerance of pathogenic bacteria to chemical fungicides, further threatening the safe production of tomato facilities (Zuo et al., 2024). Therefore, it is urgent to develop an efficient and ecologically safe method to control soil-borne diseases such as plant tomato wilt. The use of probiotics, as well as bio-antimicrobial materials derived from natural material ingredients, offers a promising environmentally friendly approach to mitigating the occurrence of soil-borne diseases.

The survival and colonization of beneficial microorganisms in the plant rhizosphere can promote growth and prevent diseases, serving as an important measure for controlling soil-borne diseases (Tyagi et al., 2024). In studies on induced systemic resistance, the most frequently cited beneficial microorganisms are *Trichoderma, Bacillus, and Pseudomonas*, and the most commonly used plant is *Solanum lycopersicon* L. (Vieira et al., 2024). After the application of beneficial microorganisms, the original community composition and structure of soil microorganisms are affected, and the complexity of the microbial co-occurrence network is significantly increased (Kong et al., 2019). The niche competition of beneficial microorganisms in the crop rhizosphere, especially at ecological sites susceptible to pathogen infection like crop root wounds, can significantly reduce the infection effect of pathogenic fungi such as *Fusarium oxysporum* (Iida et al., 2022). Additionally, some beneficial microorganisms can enhance plant disease resistance through metabolites (Kashyap et al., 2022; Manzar et al., 2024). For example, *Klebsiella* M6 can stimulate mango growth and induce plant systemic resistance by secreting IAA and hydrogen cyanide (HCN) (Kumar et al., 2021). Although probiotics play a significant role in promoting growth and preventing disease, it is difficult for beneficial microorganisms to survive and colonize in the plant rhizosphere. Thus, improving the survival and colonization of beneficial bacteria is of great importance.

Humic acids (HAs) are a macromolecular organic substance widely found in nature and is regarded as an effective biological modifier (Muscolo et al., 2013; Ibrahim and Ramadan, 2015; Qiu et al., 2024). HAs possess an abundant micropore structure, a large specific surface area, and exhibit a good adsorption effect on various substances (Li, 2002). It can be serve as a carrier of biocontrol bacteria and promote the adsorption of biocontrol bacteria (Li, 2002). In addition, HAs have excellent dispersion performance, which can reduce the surface tension of the solution and has the characteristics of dispersing and emulsifying polymer substances. They also have good surface activity, which can improve the contact between biocontrol bacteria and pathogenic bacteria and enhance the control efficiency (Sun, 2017). The combined application of humic acid and *B. subtilis* can significantly increase the yield of cucumber (Jiang et al., 2022b). HAs can improve the nutrient utilization efficiency of plants by promoting physiological effects such as carbon and nitrogen metabolism and secondary metabolic processes of plants. At the same time, it can also induce systemic resistance in plants, facilitate the growth of beneficial microbes, increase the complexity of microbial network and inhibit the relative abundance of phytopathogenic fungi (Wei et al., 2021; Jiang et al., 2022a; Jiang et al., 2022b). Given the numerous functions of HAs as described above, it may have a good effect as an additive of bio-antimicrobial agent.

Chitosan possesses excellent biological properties and shows effectiveness against soil-borne pathogens (Badawy and Rabea, 2012). Studies have demonstrated that chitosan can inhibit the growth of pathogens and improve the structure and composition of microbial communities in the inter-root soil (da Silva et al., 2021). However, the solubility of chitosan significantly limits its antibacterial activity. Enhancing the cationic properties of chitosan can improve its solubility and enhance its antimicrobial activity (Hoque et al., 2016; Sahariah et al., 2018). Theoretically, the combined application of HAs and chitosan can achieve improved antimicrobial activity as chitosan is capable of generating positively charged cation groups through HAs. Previous studies have shown that the combined application of humic acid and chitosan can produce co-inhibition effect on *Alternaria solani* growth, and this co-inhibition effect is associated with downregulated genes expression correlated with mycelial growth (Qiu et al., 2024). Under Laboratory culture condition, the community structure of soil bacteria and fungi is changed with co-application of humic acid and chitosan. In addition, the relative abundance of pathogens in the soil is decreased, while the relative abundance of beneficial bacteria is increased (Bao et al., 2022).

Based on the foregoing analysis, the combination of bio-antimicrobial materials, namely humic acid, chitosan, and probiotics, are hypothesized to hold crucial potential in promoting growth and preventing diseases. Nevertheless, the actual impact of the combined application of HAs, chitosan, and probiotics on tomato growth and soil-borne pathogens remains ambiguous. In this study, HAs, chitosan, and *B. subtilis* were combined to form a humic acid compound preparation (HBC), which was then applied to the soil for a pot experiment. The effects of HBC on tomato growth and soil-borne pathogens were explored by examining the growth index, disease index, structural changes in the rhizosphere soil microbial community, and root exudate composition of tomatoes. The research results offer a reference for the green control of soil-borne diseases in vegetable fields and the safe production of vegetables.

## 2 Materials and methods

### 2.1 Materials

Surface soil (0–20 cm) of a vegetable field was collected from the Huacheng Vegetable Cooperative in Wufeitang Village, Lishui District, Nanjing City, Jiangsu Province (located at 119°0′15″E, 31°30′25″N). The plots at the sampling sites had been continuously planted with tomatoes for more than 3 years and had experienced severe blight in the previous season (with a disease index ranging from 40 to 60% at the soil collection location). The basic physicochemical properties of the collected soil are as follows: pH value is 6.47; total nitrogen content is 1.88 g·kg^−1^; total phosphorus content is 1.90 g·kg^−1^; total potassium content is 11.40 g·kg^−1^; alkaline hydrolyzed nitrogen is 124.12 mg·kg^−1^; available phosphorus is 479.05 mg·kg^−1^; available potassium is 371.10 mg·kg^−1^; exchangeable acid is 0.19 cmol·kg^−1^; and soluble salt is 6.09 g·kg^−1^. Humic acids (HAs) were extracted from swine manure sourced from Jiangxi. The swine manure utilized for HAs extraction was air-dried and sieved to a particle size of ≤0.25 mm. The sample was extracted using 0.1 M Na_4_P_2_O_7_-NaOH with a weight-to-volume ratio (w/*ν*) of 1:10. Further details can be found in Wu et al. (2016). Purified HAs powders obtained by freeze-drying (EYELA FDU-1200, Japan) were used to measure fungistatic activity. The chitosan (CS) was obtained from Shanghai Macklin Biochemical Co., Ltd. (Shanghai, China). The *B. subtilis* strain was purchased from Agricultural Culture Collection of China (ACCC). The *B. subtilis* was cultured overnight with LB (Luria-Bertani) medium.

### 2.2 Pot experiment

A pot experiment was performed in a greenhouse to evaluate the effects of three types of bio-antimicrobial materials, namely humic acid, *B. subtilis*, and chitosan, on tomato growth and disease. A total of seven treatments were meticulously designed: control (CK), *B. subtilis* alone (BS), humic acid alone (HA), chitosan alone (CS), humic acid combined with *B. subtilis* (HB), humic acid combined with chitosan (HC), and the combination of humic acid, *B. subtilis*, and chitosan (HBC). For each treatment, four pots were utilized.

In the pot experiment, based on our previous research (Bao et al., 2022; Qiu et al., 2024), the addition amounts of humic acid, chitosan, and *B. subtilis* in soil were precisely set at 0.5 g·kg^−1^, 2 g·kg^−1^, and 10^6^ colony-forming units per gram (cfu·g^−1^), respectively. Prior to sowing, thoroughly mix the calculated the doses of humic acid, chitosan, and *B. subtilis*, then evenly scatter the mixture into flowerpots containing 1.3 kilograms the soil. Use tools such as a small shovel to stir evenly to ensure that the soil is in full contact with the additives. The disinfected tomato seeds of the Cooperative 903 variety were induced to germinate by being covered with moist gauze in a petri dish under an ambient temperature ranging approximately from 25°C to 30°C. Subsequently, eight germinated seeds were sown into each pot containing 1.3 kilograms of soil. One week after the establishment of the experiment, the plants in each pot were thinned to four. Water regularly to maintain soil water content was maintained at 60% of the water holding capacity (WHC). At the 9th week, the end of the flowering and fruiting period, the wilt disease index of tomatoes was thoroughly investigated, and the plants were harvested. Meanwhile the rhizosphere soil and root secretions of tomatoes were carefully collected, and both the plant height and the fresh weight of the plants were measured with precision.

### 2.3 Disease index

In the pot experiment, tomatoes had experienced severe wilt. Tomato wilt is a typical soil-borne disease, mostly caused by coinfection of pathogen complexes, such as *Fusarium brachygibbosum*, *Fusarium oxysporum and Ralstonia solanacearum* (Liu et al., 2024). Regarding the tomato disease index, in this study, it was not distinguished which pathogen caused the wilt. The wilt disease index (DI) of tomatoes under each treatment was investigated at the 9th week. The disease severity in response to wilt disease was classified into five levels according to the leaf wilting ratio: grade 0: no leaves are wilting; grade 1: less than 25% of leaves, branches and stem are wilting; grade 2: 25 to 50% of leaves, branches and stem are wilting; grade 3: 50 to 75% of leaves, branches and stem are wilting; grade 4: 75 to 100% of leaves, branches and stem are wilting. Then, the disease index (DI) was calculated according to the following formula (Fang, 1998):

DI = [∑ (The number of diseased plants in this grade × Disease grade) / (Total number of plants investigated × the highest disease grade)] × 100 (1).

### 2.4 The rhizosphere soil and root exudates collection

The methods of collecting rhizosphere soil and root exudates are as follows (Yan et al., 2024): On the premise of ensuring the integrity of plant roots, tomato plants were taken out of the pot. The soil attached to the roots was gently shaken off by shaking the root method. The rhizosphere soil with close adhesion was collected and placed in a sterile bag and stored at −20°C. After the collection of tomato plants was completed, the tomato roots were repeatedly rinsed with ultra-pure water. Then, all four plants collected from each pot were placed pot into a 500 mL plastic bottle wrapped with tin foil for shading. 200 mL ultra-pure water was added to ensure that the tomato roots were fully immersed in water while the stems and leaves were exposed outside the bottle to continue photosynthesis. After standing culture for 24 h, the plants were removed. The obtained solution was extracted by ethyl acetate reflux extraction method. The obtained sample was evaporated by rinsing with 2 mL methanol and stored at −20°C. Substance identification and metabolic pathway annotation were performed by ultra-high performance liquid chromatography-mass spectrometry (UHPLC–MS) by Shanghai Biozeron Biological Technology Co., Ltd.

### 2.5 Soil DNA extraction, PCR and processing of the high throughput sequencing data

The total genomic DNA of rhizosphere soil was extracted using the FastDNA SPIN Kit for soil (MP Biomedicals, Santa Ana, CA, USA) in according to the manufacturer’s instructions. The extracted soil DNA was dissolved in 60 μL of Tris-EDTA buffer, quantified with a spectrophotometer and stored at −20°C until further use. PCR amplification was carried out with primer sets 341F (CCTACGGGNGGCWGCAG) and 806R (CCTACGGGNGGCWGCAG) targeting the V3-V4 region of the bacterial 16S rRNA gene, and ITS5 (5′- GGAAGTAAAAGTCGTAACAAGG-3′) and ITS2 (5′-GCTGCGTTCTTCATCGATGC-3′) targeting the fungal ITS gene. The amplifed PCR products were sequenced on the Illumina MiSeq PE300 platform (Illumina Inc., USA). QIIME2 (version 2020.8)^1^ (Bolyen et al., 2019) was utilized for analyzing raw sequencing data. Reads with length less than 200 bp or with average quality scores less than 25 were removed, resulting in 1,875,767 high-quality sequences of the bacterial 16S rRNA gene and 1,876,217 high-quality sequences of the fungal ITS gene. Clustering of sequences into operational taxonomic units (OTUs) at a 97% nucleotide similarity level was performed using UCLUST. Generally, the sequences were clustered into 14,063 bacterial amplicon sequence variants (ASVs) and 932 ASVs after excluding singletons and rarefying them to an even sequencing depth (100,000 sequences per sample for bacteria and 50,000 sequences per sample for fungi). The 16S rRNA and fungal ITS gene sequences were annotated by Silva database version 138^2^ and the UNITE database version v8.3^3^, respectively. The raw 16S rRNA sequences and ITS sequences were submitted to the NCBI Sequence Read Archive (SRA) under the accession numbers SRP 14610143.

### 2.6 Data analysis

The fresh weight, plant height, and disease index of tomato under different treatments were analyzed using IBM Statistical Product and Service Solutions (SPSS, version 25.0) via one-way analysis of variance (ANOVA) with a significance level of *p* < 0.05. The data were visualized using Origin Pro 2024b. R 4.1.3 software was used to statistical analyses. Principal coordinate analysis (PCoA) on the ASVs and root exudates was analyzed using the “ggplot2,” “ade4,” and “vegan” package in R software. The relative abundances of fungi and bacteria at different taxonomic levels and the relative abundances of root exudates were calculated using the “ggplot2” package in R software, respectively. The *α*-diversity of microbial communities and root exudates among different treatment samples was analyzed using the “vegan” package in R software. The differential ASV in microbial communities were screened using the “edgeR” package in R software. The Spearman correlation coefficients between ASVs with relative abundance greater than 0.01% were calculated, and the *p* values were corrected using the false discovery rate (FDR) method. Only the highly significant (*p* < 0.001) mutual relationships were retained. The co-occurrence networks, subnetworks of different samples, and pathogen-beneficial microbe networks were constructed using the “igraph” package in R software. The topological characteristics of the subnetworks in each treatment were statistically analyzed, and the co-occurrence network diagrams were visualized using the Gephi program (version 0.9.3).

## 3 Results

### 3.1 Variations of tomato growth and disease severity under different treatments

**Figure 1:**
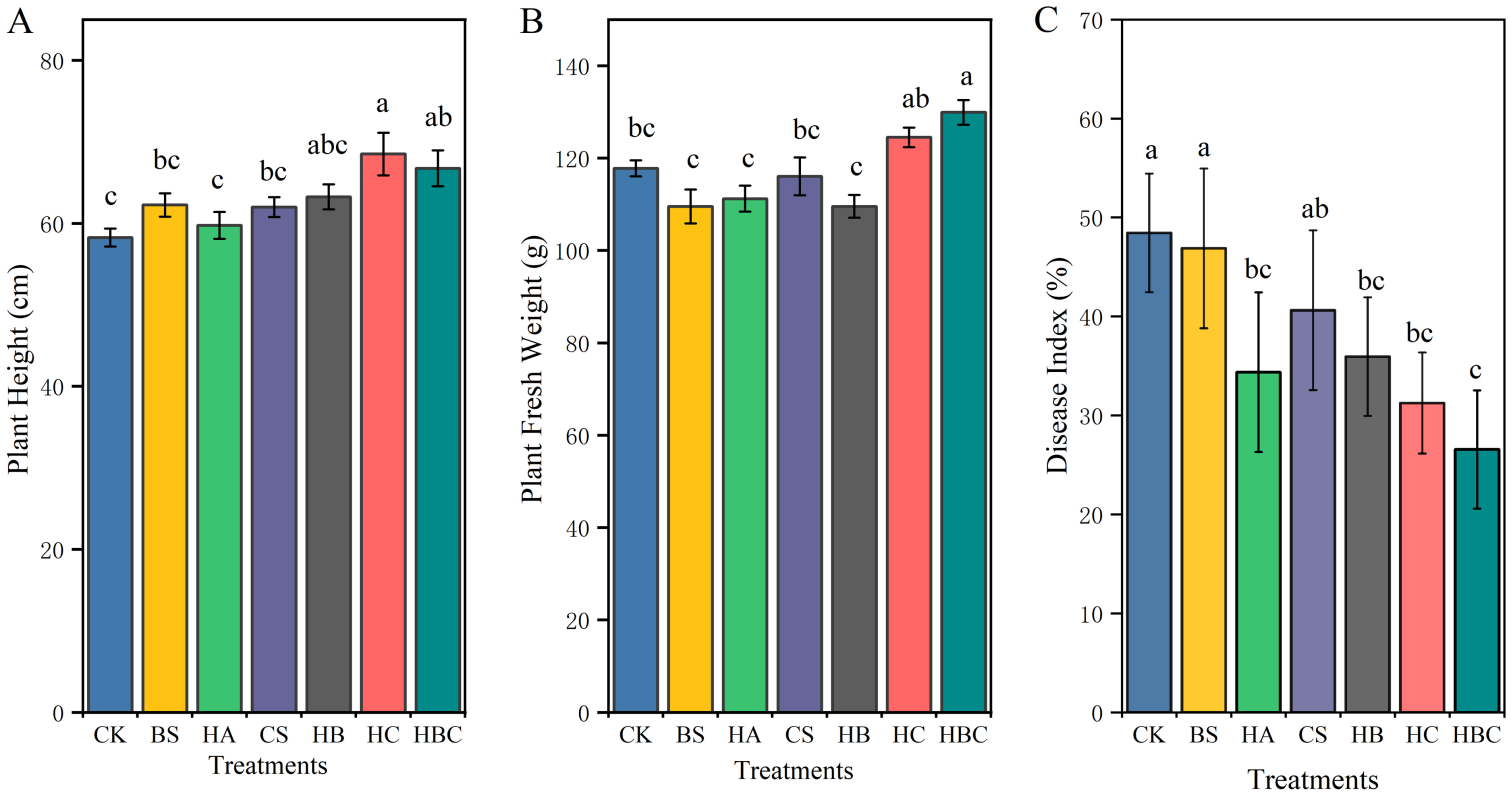
Plant height **(A)**, fresh weight **(B)** and disease severity **(C)** of tomato under different treatments. Error bars represent the standard deviations of the mean of four independent cultures. CK, control; BS, *B. subtilis*; HA, humic acid; CS, chitosan; HB, humic acid and *B. subtilis*; HC, humic acid with chitosan; HBC, humic acid, *B. subtilis* and chitosan. Different letters indicate a significant difference (Duncan’s test, *p* < 0.05).

In comparison with the CK treatment, the HC and the HBC treatments significantly increased plant height of tomatoes (*p* < 0.05), and HBC notably enhanced the fresh weight biomass (*p* < 0.05), while there was no significant difference observed under other treatments (Figures 1A,B). Additionally, in contrast to the CK treatment, the HA, HB, HC and HBC treatments significantly reduced the disease index of tomato wilt (*p* < 0.05). Among these, the disease index under the HBC treatment was the lowest, being 45.1% lower than that of CK (Figure 1C). The disease index in HA, HB and HC was 29.0, 25.8 and 35.5% lower than that of the CK treatment, respectively. There was no significant difference (*p* > 0.05) in the disease indexes among HA, HB, HC and HBC treatments.

### 3.2 Composition and diversity of soil microbial Community in Tomato Rhizosphere

**Figure 2:**
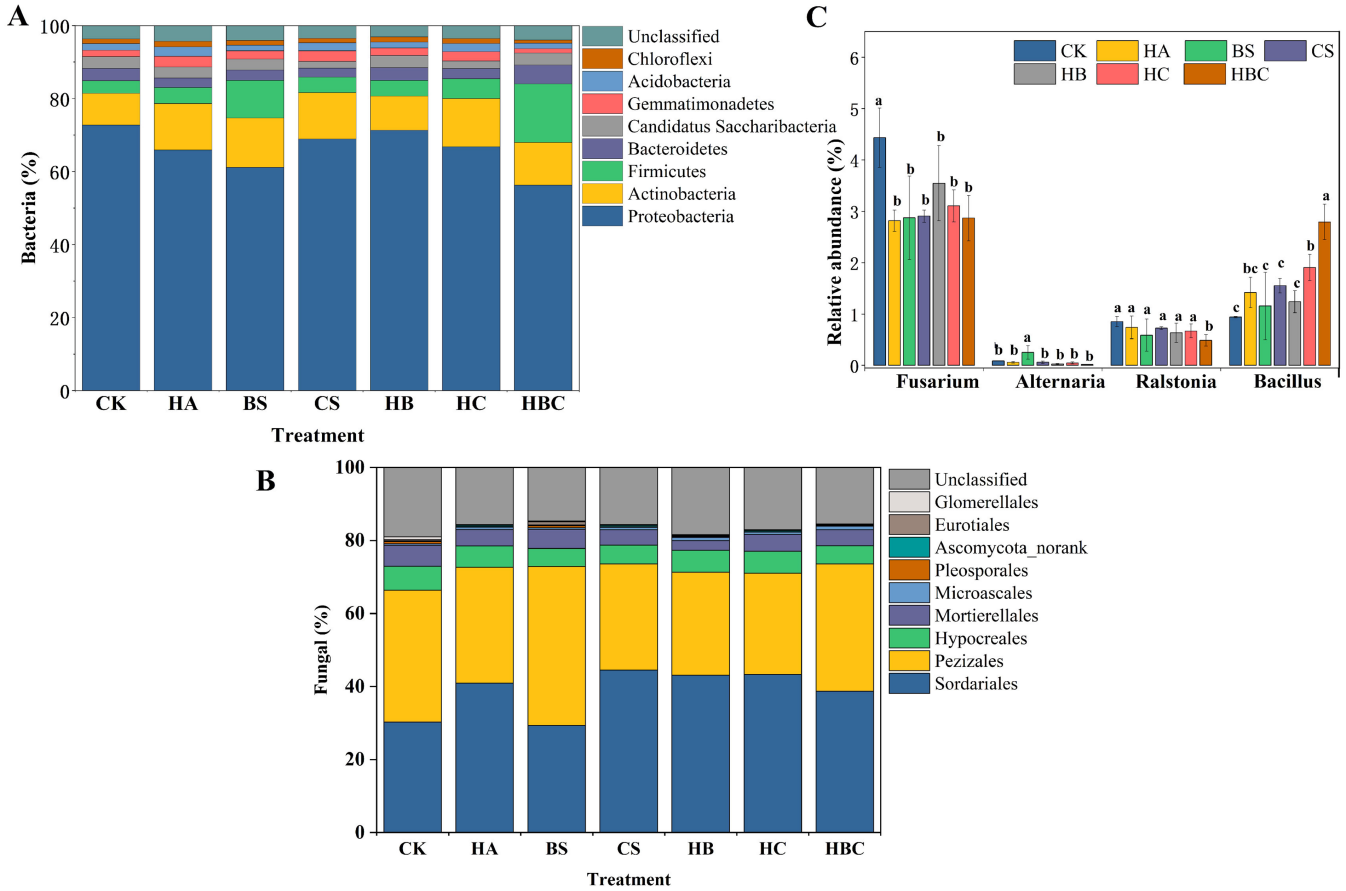
Composition of bacterial **(A)** and fungal **(B)** community and relative abundance (%) of pathogenic/beneficial microorganisms **(C)** in tomato rhizosphere under different treatments. Error bars represent the standard deviations of the mean of four independent cultures. CK, control; BS, *B. subtilis*; HA, humic acid; CS, chitosan; HB, humic acid and *B. subtilis*; HC, humic acid with chitosan; HBC, humic acid, *B. subtilis* and chitosan. Different letters indicate a significant difference (Duncan test, *p* < 0.05).

The relative abundance of bacterial communities was calculated at the genus level. The results indicated that the composition of bacterial communities in each treatment was similar (Figure 2A). *Sphingomonas, Sphingomicrobium, Cronobacter, Candidatus Saccharimonas, Enterobacteriaceae_Unclassified, Gemmatimonas, Escherichia, Bacillus, Neobacillus, Bartonella, Acinetobacter and Mesorhizobium* were the dominant bacteria in tomato rhizosphere under different treatments. Compared with the CK treatment, with the addition of bio-antimicrobial materials, the relative abundances of *Gemmatimonas, Escherichia, Neobacillus, Bacillus* and *Acinetobacter* increased, while the relative abundances of *Sphingomonas, Bartonella* and *Mesorhizobium* decreased. These effects on bacterial communities were more pronounced in the HBC treatment. At the genus level, the fungal communities in each treatment were mainly composed of *Ascobolus, Humicola, Botryotrichum, Pseudaleuria, Mortierella, Fusarium, Ovatospora*, and *Purpureocillium*, among which *Ascobolus* and *Humicola* were dominant. Compared to the CK treatment, the addition of bio-antimicrobial materials increased the relative abundances of *Humicola, Botryotrichum, Ovatospora* and *Purpureocillium*, and decreased the relative abundances of *Mortierella* and *Fusarium* (Figure 2B).

The relative abundances of *Alternaria, Fusarium* and *Ralstonia* in tomato rhizosphere soil were analyzed (Figure 2C). *Fusarium* and *Ralstonia*, which are typical soil-borne pathogens, can cause tomato wilt. It was found that compared with the CK treatment, the relative abundances of *Fusarium* significantly decreased in all treatments with bio-antimicrobial materials. The relative abundances of *Ralstonia* significantly decreased (*p* < 0.05) in the HBC treatment, while there was no significant difference in other treatments. The bio-antimicrobial materials had no significant effect on *Alternaria. Bacillus,* a typical beneficial bacterium in soil, was also analyzed. It was found that the relative abundance of *Bacillus* significantly increased (*p* < 0.05) in HC and HBC treatments compared with the CK treatment.

**Figure 3:**
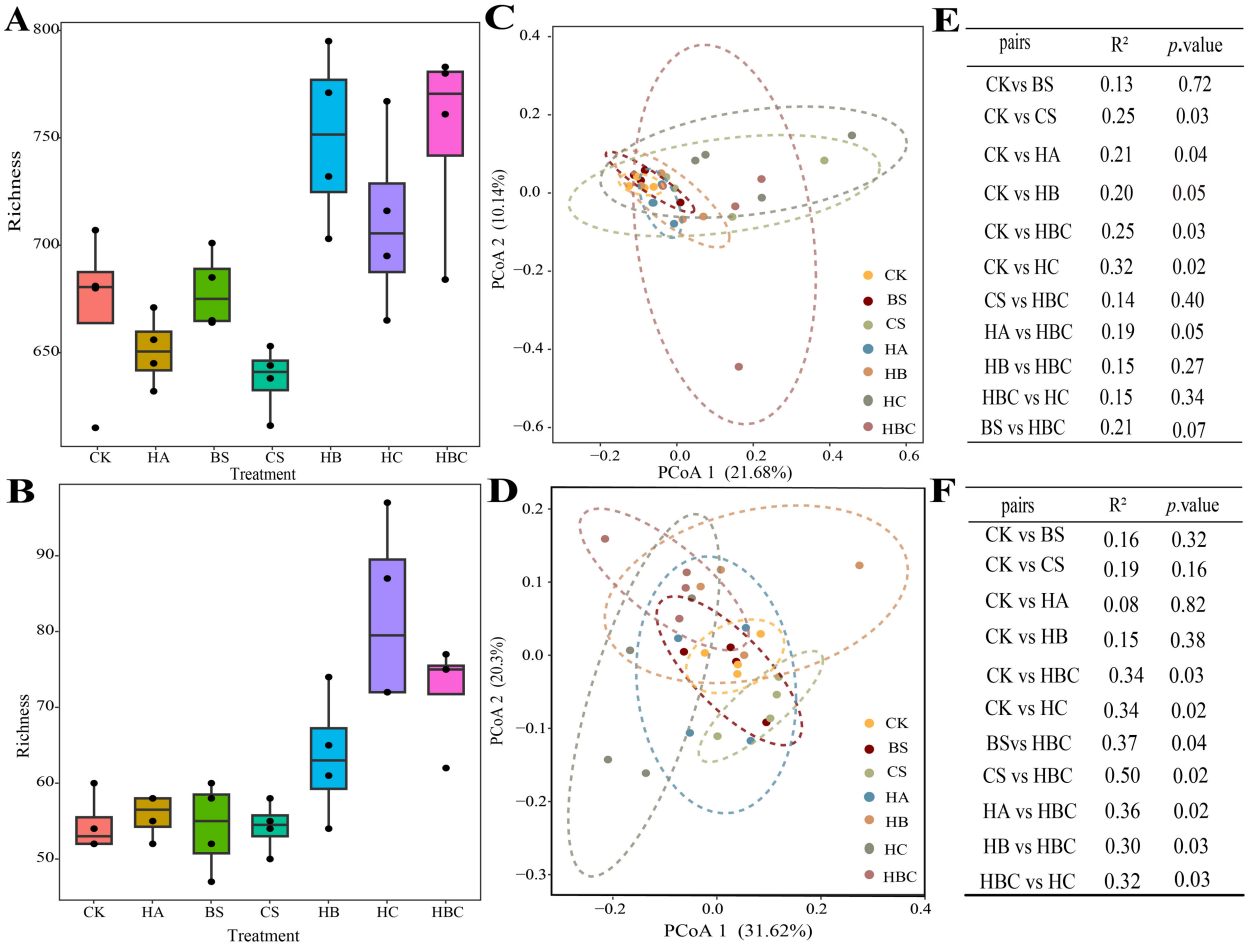
Diversity, clustering difference and significance of bacterial **(A,C,E)** and fungal **(B,D,F)** communities in tomato rhizosphere under different treatments. CK, control; BS, *B. subtilis*; HA, humic acid; CS, chitosan; HB, humic acid and *B. subtilis*; HC, humic acid with chitosan; HBC, humic acid, *B. subtilis* and chitosan.

The microbial richness of tomato rhizosphere soil was calculated (Figures 3A,B). Compared with the CK treatment, the richness of bacterial and fungal community in the HBC treatment significantly increased (*p* < 0.05). This indicated that the humic acid compound preparation improved the diversity of microbial community in tomato rhizosphere soil.

Principal coordinate (PCoA) analysis was conducted to explore the clustering differences of tomato rhizosphere microbial communities under different treatments. The results showed that the first and second axes explained 21.68 and 10.14% variation in the soil bacterial community, respectively. Moreover, the bacterial community structure in CS, HA, HC and HBC was significantly different from that in CK (*p* < 0.05) (Figures 3C,E). The first and second axles explained 31.62 and 20.30% of the variation in the soil fungal community, respectively. And the fungal community structure in HC and HBC was significantly different from that in CK (*p* < 0.05) (Figures 3D,F). The results demonstrated that the humic acid compound preparation (humic acid, chitosan and *B. subtilis*) had significant effect on the tomato rhizosphere microbial community structure.

### 3.3 Diversity and composition of root exudates of tomato

**Figure 4:**
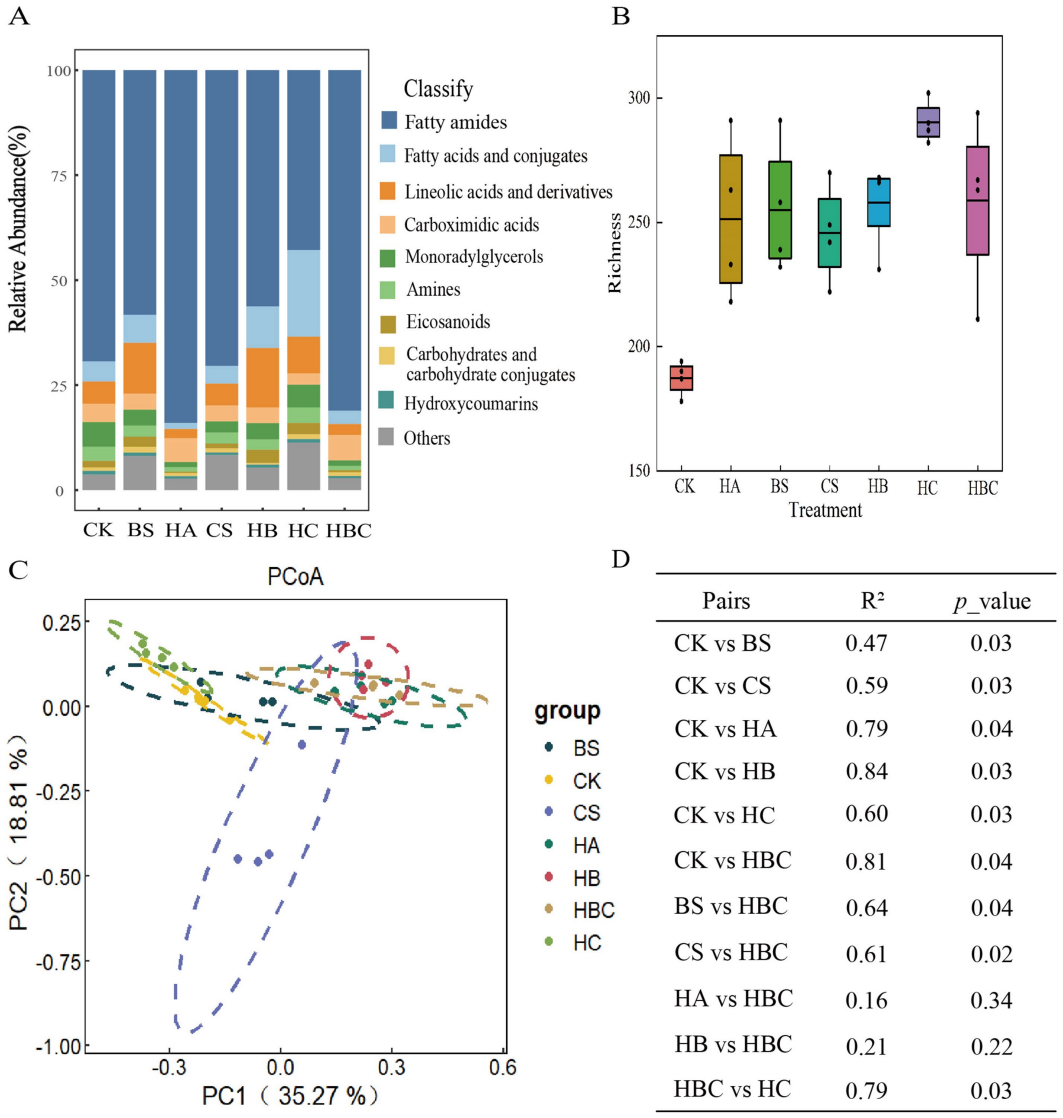
Composition **(A)**, diversity **(B)**, clustering difference **(C)** and Significance **(D)** of tomato root exudates under different treatments. CK, control; BS, *B. subtilis*; HA, humic acid; CS, chitosan; HB, humic acid and *B. subtilis*; HC, humic acid with chitosan; HBC, humic acid, *B. subtilis* and chitosan.

The relative abundance of tomato root exudates under different treatments was analyzed. The results revealed that compared with the CK treatment, the relative abundance of Carboximidic acids and fatty amides increased, while the Lineolic acids and derivatives, Fatty acids and conjugates, Monorady lglycerols, and Amines and Eicosanoids compounds decreased in the HA and HBC treatments (Figure 4A). The richness of tomato root exudates was calculated (Figure 4B). It was found that the richness of root exudates increased under bio-antimicrobial materials treatment compared with the CK treatment, indicating that the bio-antimicrobial materials improved the diversity of tomato root exudates.

Principal coordinate (PCoA) analysis was conducted to explore the clustering differences of tomato root exudates under different treatments. The results showed that the first and second axes explained 35.27 and 18.81% of the variation in root exudates, respectively. Moreover, the root exudates structure under bio-antimicrobial materials treatment was significantly different from that in the CK treatment (*p* < 0.05) (Figures 4C,D).

### 3.4 Co-occurrence network analysis between rhizosphere microbial community and root exudates of tomato

In this study, the topological characteristics of the co-occurrence network between the rhizosphere microbial community and root exudates of tomato under different treatment were analyzed (Table 1). It was found that the connectivity, average degree and average path length of the networks under each treatment showed no significant differences, indicating that the bio-antimicrobial materials had no significant effect on the interaction between the rhizosphere microbial community and that of tomato.

**Table 1:** Topological characteristics of microbe-exudate interaction network under different treatments.

Treatment	Connectivity	Average degree	Average path length
CK	0.02 ± 0.00 a	4.88 ± 0.55 b	2.97 ± 0.18 a
BF	0.02 ± 0.00 a	7.01 ± 0.97 ab	2.96 ± 0.04 a
CS	0.03 ± 0.00 a	9.30 ± 1.16 ab	2.85 ± 0.32 a
HA	0.03 ± 0.00 a	5.93 ± 1.39 b	2.94 ± 0.43 a
HC	0.04 ± 0.01 a	16.06 ± 6.32 a	2.50 ± 0.07 a
HB	0.02 ± 0.00 a	6.74 ± 1.26 ab	3.16 ± 0.10 a
HBC	0.02 ± 0.00 a	5.61 ± 1.52 b	2.73 ± 0.24 a

Different letters in the same column indicate significant differences (Duncan’s test, *p*<0.05).

**Figure 5:**
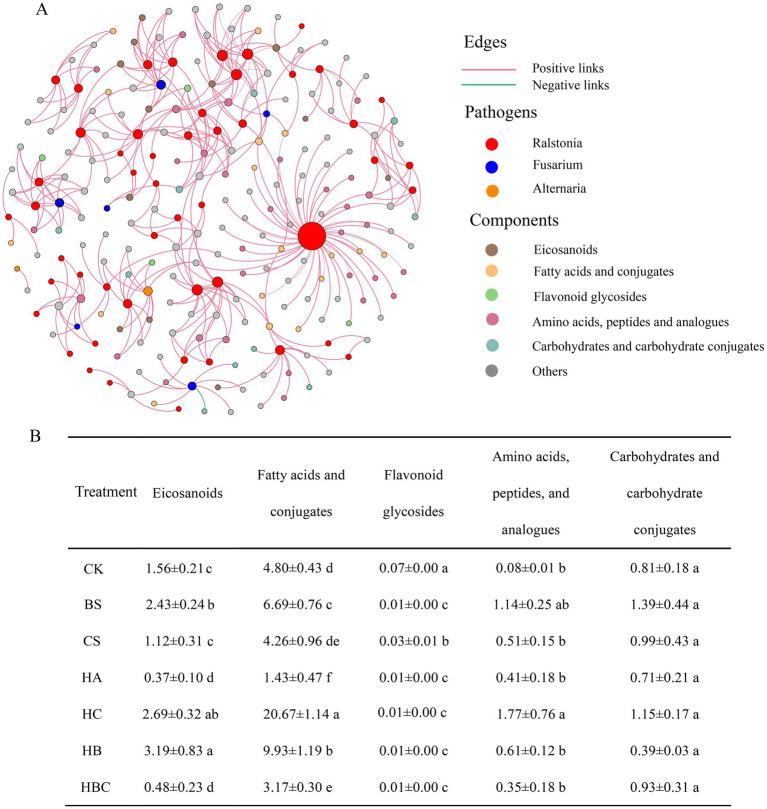
The interaction network between rhizosphere main pathogenic microorganisms and root exudates **(A)**; Relative abundance of exudates under each treatment (%) **(B)** CK, control; BS, *B. subtilis*; HA, humic acid; CS, chitosan; HB, humic acid and *B. subtilis*; HC, humic acid with chitosan; HBC, humic acid, *B. subtilis* and chitosan. Different letters indicate a significant difference (Duncan’s test, *p* < 0.05).

To further explore the antimicrobial mechanism of bio-antimicrobial materials, the relationship between root exudates and three typical pathogens of *Alternaria, Fusarium* and *Ralstonia* was investigated. The subnetwork of pathogens and root exudates associated with them were separated from the co-occurrence network (Figure 5). It was shown that root exudates were mostly positively correlated with pathogens. Among the root exudates, Eicosanoids, Fatty acids and conjugates, Flavonoid glycosides, Amino acids, peptides, and analogs of Carbohydrates and carbohydrate conjugates were highly connected to pathogens. The abundances of Eicosanoids, Fatty acids and conjugates and flavonoid glycosides decreased significantly in the HA and HBC treatments. The results indicated that humic acid and humic acid compound preparation (HBC) had an effect on the content of tomato root exudates.

## 4 Discussion

The combined application of bio-antimicrobial materials humic acid, chitosan, and *B. subtilis* (HBC) represents a significant advancement in the realm of sustainable agriculture, particularly in promoting plant growth and combating soil - borne diseases. This study delves deeper into the underlying mechanisms and far - reaching impacts of HBC to enhance the understanding of this innovative approach.

### 4.1 Impact on plant growth and disease inhibition

In this study, it was found that under HBC treatment, tomato height and fresh weight increased significantly, and the disease index of tomato wilt was notably reduced. This indicated that the combined application of humic acid, chitosan and *B. subtilis* could promote tomato growth and inhibit soil-borne diseases. Previous studies have shown that *B. subtilis*, as a well - known plant - growth - promoting rhizobacterium (PGPR), offers multiple advantages. *B. subtilis* can alleviate abiotic stress on plants by fixing nitrogen biologically, increasing phosphorus, producing iron carrier, and promoting the production of enzymes and phytohormones (Anguiano Cabello et al., 2019; Jabborova et al., 2024; Rathod et al., 2024). *B. subtilis* not only promotes overall plant growth but also primes the plant’s defense mechanisms against pathogens. For example, *B. subtilis* has been used to suppress black spot disease of ‘Korla’ fragrant pear fruit caused by fungal pathogen and elicit systemic resistance in tomato plants against wilt pathogens (Akram et al., 2021; Wang et al., 2023). This ability to confer multi - stress tolerance is likely to contribute to the improved growth of tomatoes under HBC treatment.

Chitosan is a natural elicitor used for stimulating plant growth and inducing plant defense (Suwanchaikasem et al., 2024). Chitosan can increase the content of ascorbic acid and glutathione, the biomass of maize, and enhance drought resistance (Liu et al., 2023). Moreover, chitosan can form a physical barrier on the plant surface, preventing the entry of pathogens. When combined with other biocontrol agents like *B. subtilis*, this barrier - forming ability may be further enhanced, providing a more robust defense against soil - borne diseases. The combination of chitosan and other biocontrol agents such as beneficial bacteria and fungi can be used to manage plant yield and diseases (Agbodjato et al., 2016; Suwanchaikasem et al., 2024).

In addition, HAs can increase rhizosphere population and chemotaxis, bacteria attachment and survival on the plant surface, as well as endophytic colonization, enhance nutrient use efficiency, aid assimilation, and promote plant growth (Olivares et al., 2017). Humic acids and *H. seropedicae* can increase growth by modulating the content of organic acids in leaf tissue and attenuating the symptoms of the bacterial spot (da Silva et al., 2021). The combination of humic acid and chitosan can produce a co-inhibition effect on pathogen *Alternaria solani* growth (Qiu et al., 2024).

In this study, the combined application of HBC had a more pronounced effect on tomato growth and disease inhibition compared to individual treatments. This synergistic effect may be attributed to the complementary actions of these components. Humic acid can serve as a carrier for *B. subtilis*, facilitating its attachment and colonization in the rhizosphere. The presence of chitosan may enhance the antimicrobial activity of *B. subtilis* by altering the cell - wall permeability of pathogens (Badawy and Rabea, 2012). Together, they create a more conducive environment for tomato growth, reducing the disease index of tomato wilt by 45.1% and increasing plant height and fresh weight significantly.

### 4.2 Influence on soil microbial communities

The structural and functional characteristics of soil microbial communities play a crucial role in regulating soil health and plant growth. Microbiomes significantly enhance plant health by facilitating nutrient accessibility, promoting growth under stresses conditions, providing a natural defense against diseases and pests, and improving soil quality (Kwak et al., 2018; Gachara et al., 2024). The unbalanced proliferation of pathogens and other microorganisms in tomato rhizosphere soil can lead to the occurrence of tomato diseases. Key beneficial microbial groups can inhibit the growth of many pathogens and effectively control the occurrence of soil-borne diseases (van der Voort et al., 2016). HBC treatment led to significant changes in the relative abundances of various microbial groups in the tomato rhizosphere.

In this study, with the addition bio-antimicrobial materials, the increase in the relative abundance of *Gemmatimonas, Neobacillus, Bacillus*, *Acinetobacter,* and *Humicola* is a key finding. *Gemmatimonas* was positively correlated with crop yield, which has been associated with the degradation of complex organic matter in the soil, releasing nutrients that are readily available to plants (Zhang et al., 2022). Its increased abundance in the HBC treatment may contribute to improved soil fertility and plant nutrient uptake. *Neobacillus* has shown remarkable potential in controlling plant wilt diseases and other plant wilt diseases. It can produce extracellular enzymes such as chitinases and glucanases, which can break down the cell walls of pathogenic fungi like *Rhizoctonia solani* and *Fusarium oxysporum.* Moreover, as *Neobacillus* has the function of protease production, which may help in the degradation of proteins in the soil, making nitrogen more accessible to plants, reducing the use of fertilizer and improving crop yield (Tan et al., 2024). *Bacillus,* which are typical beneficial bacteria, play an important role in plant growth and resistance to pathogens (Kashyap et al., 2021; Vieira et al., 2024). *Acinetobacter* can be used as biofertilizer in agricultural production. Patel et al. (2022) found that Acinetobacter can improve the growth of sugarcane under salinity stress by producing IAA, soluble phosphate, potassium and zinc, and fixating of atmospheric nitrogen. *Humicola* has biological protective effect and growth-promoting effect. Studies have shown that *Humicola* can have antimicrobe activity against many plant pathogens such as fungi and bacteria by secreting some secondary metabolic substances, such as auxin and aloe saponin II (Moghaddam et al., 2020).

Conversely, the relative abundances of pathogenic microorganisms such as Sphingomonas and Fusarium decreased under HBC treatment. *Fusarium* often causes wilt in plants, while *Sphingomonas* can cause bacterial white leaf blight in the plant *Paliurus spina-christi* (Deldavleh et al., 2013). Additionally, in HBC, the relative abundance of *Bacillus* increased significantly, the relative abundance of *Fusarium* and *Ralstonia* significantly decreased. HBC had effect on both fungal and bacterial pathogen suppression. It suggests that the combined action of HBC components may disrupt the growth and survival of these pathogens. Possibly because *B. subtilis* produces antimicrobial substances, combinated with the allelopathic effects of humic acid and chitosan, it may inhibit the growth and reproduction of these pathogens. The significant increase in the diversity of fungal and bacterial communities under HBC treatment is also noteworthy. A more diverse microbial community is generally more stable and resilient, which can better resist pathogen invasion. The presence of a diverse range of beneficial microorganisms can out - compete pathogens for resources, occupy ecological niches, and prevent pathogen colonization. This positive regulation of the microbial community structure by HBC enhances the soil’s ability to suppress diseases and support plant growth.

### 4.3 Regulation of root exudates

Root exudates play a significant role in regulating the structure and function of rhizosphere microorganisms. The HBC treatment had a profound impact on the composition and secretion of tomato root exudates. The increase in the diversity of root exudates under HBC treatment is a significant finding. Studies have shown that carboximidic acids compounds can exhibit good broad-spectrum antibacterial activity, with an antibacterial rate reaching 81.2% *in vitro*. The combined application of carboximidic acids compounds and phosphate fertilizer can improve the utilization efficiency of phosphorus, promote the absorption of nutrients and increase the productivity of plants (Dang et al., 2023). In the context of HBC, this may lead to enhanced nutrient uptake by tomatoes, promoting their growth.

Conversely, the content of allelopathic compounds such as phenolic acids decreased under HBC treatment. Phenolic acids are known to have autotoxic effects on plants and can disrupt the balance of the rhizosphere microbial community. They can affect the composition of soil microflora and physiological metabolism, induce the growth of soil-derived pathogenic fungi and toxin production, which in turn affects plant growth and health (Chen et al., 2014; Wu et al., 2015; Zhao et al., 2018; Jin et al., 2020). They have an inhibitory effect on the growth of rhizosphere microorganisms and can inhibit the activity of extracellular enzymes (such as urease and sucrase, etc.) of soil microorganisms (Cui et al., 2022). For example, the secretion of rho-hydroxybenzoic acid/phthalic acid and vanillin reduced the number of beneficial rhizosphere bacteria such as *Bacillus*, *Streptomyces* and *Lysobacter*, leading to an unbalanced rhizosphere microbial community structure (Zhou and Wu, 2018; Hua et al., 2019; Liu et al., 2022). By reducing the secretion of these allelopathic compounds, HBC treatment helps to maintain a healthy rhizosphere microbial community.

The co - occurrence network analysis revealed that the contents of eicosanoids, fatty acids and conjugates, and flavonoid glycosides, which were positively correlated with pathogen communities, decreased in root exudates under HBC treatment. This may indicate that HBC can effectively disrupt the communication and interaction between root exudates and pathogens. It is possible that the components of HBC interfere with the signaling pathways that regulate the production and secretion of these pathogen - associated compounds in the roots.

In conclusion, the combined application of humic acid, chitosan, and *B. subtilis* (HBC) exerts a multifaceted influence on tomato growth and disease prevention and control. It promotes plant growth through various mechanisms. It suppresses soil - borne diseases by regulating the soil microbial community structure and reducing the secretion of allelopathic and pathogen - associated compounds in root exudates. Future research should focus on further elucidating the molecular mechanisms underlying these effects and optimizing the formulation and application of HBC for more efficient and sustainable agricultural practices.

## 5 Conclusion

In this study, the combined application of humic acid, chitosan and *B. subtilis* could significantly co-promote the growth of tomato plants, co-inhibit plant diseases, increase the diversity of the soil microbial community and root exudates, and reduce the relative abundances of soil-borne fungi in tomato rhizosphere soil. Additionally, the combined application of humic acid, chitosan and *B. subtilis* also reduces the secretion of allelopathic phenolic acid compounds and inhibits the secretion of compounds positively correlated with soil-borne pathogens in tomato vegetable fields. Our study indicates the great potential of humic acid compound preparation in combating soil-borne pathogens. Subsequently, the mechanism behind the synergistic effects of humic acids, chitosan, and *B. subtilis* on disease suppression will be further explored. Additionally, field tests will be carried out to broaden its practical application.

## Data Availability

The raw 16S rRNA sequences and ITS sequences were submitted to the NCBI Sequence Read Archive (SRA) under the accession number SRP 14610143.

## Footnotes

^1^ https://qiime2.org/

^2^ https://www.arb-silva.de/

^3^ https://unite.ut.ee/
